# Secondary structure specific simpler prediction models for protein backbone angles

**DOI:** 10.1186/s12859-021-04525-6

**Published:** 2022-01-04

**Authors:** M. A. Hakim Newton, Fereshteh Mataeimoghadam, Rianon Zaman, Abdul Sattar

**Affiliations:** 1 School of Information and Communication Technology, Griffith University, Brisbane, Australia; 2 Institute of Integrated and Intelligent Systems, Griffith University, Brisbane, Australia

**Keywords:** Dihedral angle prediction, Protein structure prediction, Deep learning

## Abstract

**Motivation:**

Protein backbone angle prediction has achieved significant accuracy improvement with the development of deep learning methods. Usually the same deep learning model is used in making prediction for all residues regardless of the categories of secondary structures they belong to. In this paper, we propose to train separate deep learning models for each category of secondary structures. Machine learning methods strive to achieve generality over the training examples and consequently loose accuracy. In this work, we explicitly exploit classification knowledge to restrict generalisation within the specific class of training examples. This is to compensate the loss of generalisation by exploiting specialisation knowledge in an informed way.

**Results:**

The new method named SAP4SS obtains mean absolute error (MAE) values of 15.59, 18.87, 6.03, and 21.71 respectively for four types of backbone angles $\phi$, $\psi$, $\theta$, and $\tau$. Consequently, SAP4SS significantly outperforms existing state-of-the-art methods SAP, OPUS-TASS, and SPOT-1D: the differences in MAE for all four types of angles are from 1.5 to 4.1% compared to the best known results.

**Availability:**

SAP4SS along with its data is available from https://gitlab.com/mahnewton/sap4ss.

## Introduction

Proteins comprise amino acid (AA) sequences and fold into three dimensional (3D) structures. The *native* structure of a protein has the minimum free energy and it determines the function of the protein. The protein structure prediction (PSP) problem is to determine the native structure of a protein from its AA sequence. PSP is computationally very challenging [1]. The challenge comes from the astronomically large conformational search space and the unknown energy function involved in the folding process [2].

Proteins have *backbones* or *main chains* comprising peptide bonds that connect *C* and *N* atoms of successive AAs. AAs all have three common atoms *N*, $C^{\alpha }$, and *C* in sequence. Typically AAs can be of 20 types based on the uniqueness of the side chains that start from their $C^{\alpha }$ atoms. AA instances in a protein are called *residues*. Protein backbone structures can be represented by a series of dihedral angles $\phi _i$, $\psi _i$, and $\omega _i$. These dihedral angles are defined respectively by every four consecutive atoms from the sequence $C_{i-1}$, $N_i$, $C^{\alpha }_i$, $C_i$, $N_{i+1}$, $C^{\alpha }_{i+1}$. However, $\omega$ angles are $180^\circ$ for majority proteins [3]. AA side chains also have their own dihedral angles but they are out of scope of this work since they can be dealt with later once backbones are obtained. Nevertheless, protein backbone structures are important for both template-based and template-free PSP [2, 4].

Besides the representation method discussed above, protein backbone structures can also be represented by $C^{\alpha }$ atoms since successive $C^{\alpha }$ atoms have almost the same distance. In this case, instead of $\phi$, $\psi$, and $\omega$, two other angles $\theta$ and $\tau$ are used. Note $\theta$ and $\tau$ are respectively a planner and a dihedral angle comprising respectively three and four consecutive $C^{\alpha }$ atoms. Since multiple residues are needed to define $\theta$ and $\tau$, they could somewhat capture local structures.

In this work, we develop deep neural network (DNN) models to predict the backbone angles $\phi$, $\psi$, $\theta$, and $\tau$ for proteins. Protein backbone angle prediction (BAP) has achieved significant progress with the development of DNNs. Yet more accurate BAP is needed since errors in any angles in a protein has a cascaded effect on the entire protein structure.

In BAP, DNN variants such as stacked sparse auto-encoder neural networks [5], long short-term memory (LSTM) bidirectional recurrent neural networks (BRNNs) [6–8], Residual Networks (ResNets) [7], and DNN ensembles [7, 8] or layered iterations [9] have been used.

Input features used in BAP include very popular position specific scoring matrices (PSSM) generated by PSI-BLAST [5–7, 9–12]; 7 physicochemical properties (7PCP) [5–7, 9, 11] such as steric parameter (graph shape index), hydrophobicity, volume, polarisability, isoelectric point, helix probability, and sheet probability [13]; predicted accessible surface area (ASA) [5, 12]; hidden Markov model (HMM) profiles [7, 11, 14] produced by HHBlits [15]; contact maps [7]; and PSP19 [8].

Capturing local structures around and long range interactions between residues have been considered in BAP. Sliding windows [5, 6, 9, 12] around residues have been used in feature encoding to capture the local structures. On the other hand, entire protein sequences have been used as features [9, 11, 16] to capture long range interactions. Convolutional neural networks (CNNs) [8, 14] or LSTM-BRNNs [6, 7] have also been used to capture long range interactions.

For benchmark datasets, we refer to PISCES [17], SPOT-1D [7, 18], PDB150 [19] and CAMEO93 [20]. The first two are large with respectively 5.5K and 12.5K proteins and 1.2M and 2.7M residues. The last two are small with 150 and 93 proteins respectively and are used in testing.

Proteins locally exhibit three major secondary structure (SS) types such as helices, sheets, and coils. This three-state classification can be extended to an eight-state classification. Essentially some SS types are associated with angle ranges. For example, helices and sheets have ranges of 20 for $\phi$ and $\psi$. Because of these narrow angle ranges, BAP could be essentially viewed as a classification problem via SS type prediction, although backbone angles are actually continuous valued. Unfortunately, coils have no ranges and they are about 40% of residues in an average protein [21]. So SS prediction essentially does not make BAP trivial. SS prediction has obtained significant progress via DNN models [8, 11, 22–26] and *ab initio* methods [27]. SSpro8 [28] achieves respectively 92% and 79% accuracy on proteins with or without homologs in the Protein Data Bank (PDB).

Predicted SS types have been used as features in deep learning for BAP [5, 9, 12, 29]. Features in general in deep learning only implicitly capture problem characteristics and the neural network model then attempts to establish the unknown input-output relation but again very implicitly. Also, any machine learning method strives to achieve generality over the training examples and consequently loose accuracy in the process. While generic artificial intelligence (AI) methods could be adapted to a range of problems easily, they usually suffer from the loss of explicit problem specific knowledge. So explicit exploitation of any available knowledge is of great importance in AI. This could actually bridge the gap between the generality of the approach with the specificity of the problem. Inspired by this AI interest, we attempt to explicitly exploit predicted SS knowledge in BAP. To be more particular, for BAP, we train separate deep learning models for each SS category. This restricts the generalisation only within the specific class of training examples and thus compensates the loss of generalisation by exploiting specialisation knowledge in an informed way.

We name our new BAP method as Simpler Angle Predictor for Secondary Structures (SAP4SS), which has DNN models similar to a very recent BAP method named SAP [30]. Like SAP, our new method SAP4SS has simpler DNN models than what other recent methods such as OPUS-TASS [8] and SPOT-1D [7] have. SAP4SS uses the same fully connected neural network (FCNN) architecture as SAP does while OPUS-TASS and SPOT-1D use ensembles of LSTM-BRNNs and ResNets. SAP4SS has more features than SAP but fewer features than OPUS-TASS and SPOT-1D. While SAP has been trained on all residues, SAP4SS has separate DNN models for residues that belong to separate 3-state SS types.

On well-known benchmark datasets, SAP4SS obtains mean absolute error (MAE) values 15.59, 18.87, 6.03, and 21.71 respectively for $\phi$, $\psi$, $\theta$, and $\tau$ predictions. As a result, SAP4SS significantly outperforms existing state-of-the-art methods SAP, SPOT-1D and OPUS-TASS: the differences in MAE are from 1.5 to 4.1% compared to the best known results. The SAP4SS program along with its data is available from the website https://gitlab.com/mahnewton/sap4ss.

## Related works

Recent backbone angle prediction methods include ANGLOR [12], SPIDER [5], SPIDER2 [9], SPIDER3 [6], RaptorX-Angle [29], DeepRIN [19], NetSurfP-2.0 [14], SPOT-1D [7], OPUS-TASS [8], and SAP [30].

ANGLOR [12] predicts $\phi$ and $\psi$ angles separately, by utilising neural networks and support vector machines (SVM) [31] respectively. SPIDER [5] applies a stacked sparse auto-encoder deep neural network for predicting $\theta$ and $\tau$ angles. SPIDER2 [9] uses three iterations of the SPIDER-type models. SPIDER3 [6] applies a bidirectional recurrent neural networks (BRNN) with predicted backbone torsion angles, predicted secondary structures, and predicted solvent accessibilities as input. The predicted features are reused to train the BRNN iteratively for four times. RaptorX-Angle [29] employs a combination of clustering and deep learning for predicting $\phi$ and $\psi$ values. DeepRIN [19] utlises deep residual inception network to predict $\phi$ and $\psi$ values. NetSurfP-2.0 [14] employs large Long Short-Term Memory (LSTM) networks in BRNNs to predict $\phi$ and $\psi$ angles.

SPOT-1D uses an ensemble of 9 LSTM-BRNN and ResNets with input features PSSM, HMM, 7PCP, and contact maps. The contact maps are from SPOT-Contact [18] and are used in a sliding window fashion. However, SPOT-1D also uses entire proteins at a time as input. SPOT-1D predicts all four types of backbone angles. The output angles are predicted as trigonometric ratios. OPUS-TASS predicts only $\phi$ and $\psi$ angles with ensembles of DNNs having CNN, LSTM, and Transformer [32] layers. It has an input feature PSP19 [33] that classifies residues into rigid-body blocks. OPUS-TASS introduces a new constrained feature CSF3 [34] to describe backbone structures. OPUS-TASS uses a multi-task learning strategy [35]. SAP predicts all four types of backbone angles using a simple FCNN with sliding windows, 8-state SS predictions, PSSM, and 7PCP input features.

## Methods

In this section, we describe the deep learning models used in SAP4SS and the datasets used in experiments. These are similar to those used in SAP [30] but SAP4SS has additional input features and have separate DNN models for SS types.

### Input features

**Fig. 1:**
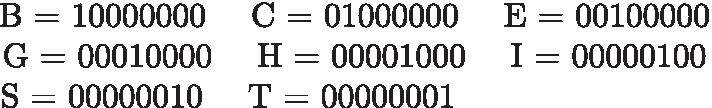
Encoding of 8-state SS predictions by SSPro8 using one-hot vectors. Exactly one bit in each bit string of length 8 has 1 in it and the other 7 bits are 0s

Like SAP, for each residue, we consider the following features: 8-state SS prediction by SSpro8 [28] where the prediction is encoded as a one-hot vector as shown in Fig. 1; 20 features from PSSM profile generated by three iterations of PSI-BLAST [10] against the UniRef90 sequence database updated in April 2018; and 7PCP. Moreover, for each residue, in SAP4SS, we additionally consider 20 residue-substitution features from HMM profile generated by HHblists [36] and 1 feature for ASA predicted by SPOT-1D [7]. These make at most 56 features for each residue but we evalute their effects on the prediction accuracy by using and not using HMM and ASA features. To capture local structures around each residue, like SAP, we consider sliding windows of size *W* where $\lfloor W/2\rfloor$ residues are after and $\lfloor W/2\rfloor$ residues are before a given residue. Although some BAP methods based on recurrent neural networks (RNN) and CNNs use entire proteins at a time, we do not do so since the effects of very long range interactions are not clear from distance based molecular dynamic forces. For each residue, for a given window size *W*, we thus have up to 56*W* input features. Based on SAP’s reported experimental results, for SAP4SS, we consider window sizes 5 and 9 only. Nevertheless, these input features are encoded either by using [0, 1] range based (shown in (1)) or Z-score based (shown in (2)) normalisation methods before feeding to the DNNs. In (1), $x_\text {min}$ and $x_\text {max}$ are the minimum and maximum values in the training set for the feature which *x* is coming from. In (2), $\mu$ and $\sigma$ are the mean and standard deviation of the values in the training set for the feature which *x* is coming from. Both in (1) and (2), $x'$ is the encoded value.

1 $$\begin{aligned} x'= & {} \frac{x - x_{\text {min}}}{x_{\text {max}}- x_{\text {min}}} \end{aligned}$$

2 $$\begin{aligned} x'= & {} \frac{x - \mu }{\sigma } \end{aligned}$$

### Predicted outputs

For each residue, SAP4SS predicts four angles $\phi$, $\psi$, $\theta$, and $\tau$ where $\theta _i$ angle is defined by $C^{\alpha }_{i-1}$, $C^{\alpha }_i$, and $C^{\alpha }_{i+1}$ while $\tau _i$ is defined by $C^{\alpha }_{i-1}, C^{\alpha }_i$, $C^{\alpha }_{i+1}$, and $C^{\alpha }_{i+2}$. Like SAP, SAP4SS predicts the angle values directly. To ensure the periodicity of $[-180^\circ , 180^\circ ]$ of the predicted angles, $360^\circ$ is subtracted from or added to any angle value greater than $180^\circ$ or less than $-180^\circ$ respectively.

### Loss functions

We use MAE as the loss function in the DNN models. We calculate absolute error $\text {AE} = \min (D, |360 - D|)$ where $D = |P - A|$, *P* is a predicted angle, and *A* is the actual angle for a residue. The mean is taken over the AEs for all residues. We ignore the angles at the beginning or at the end of the proteins, since they are not defined. The AE deals with the angle perodicity issue and is used in validation and testing as well.

### Neural networks

We follow the same neural network architecture and implementation of SAP. In summary, we use an FCNN with 3 hidden layers, each having 150 neurons. Different numbers of layers have been experimented by SAP; so we do not run similar experiments again. The number of input features to the FCNN will depend on using and not using of HMM and ASA features and the window size. The Python-based FCNN implementation uses Keras library, SGD optimiser with momentum 0.9, and kernel initialiser glorot_uniform. The learning rate is initially 0.01. Then, with three successive iterations having no improvement in the loss functions, the learning rate gets reduced by a factor of 0.5 until reaches $10^{-15}$. For the input and hidden layers, the activation function is sigmoid while it is linear for the output layer. NVIDIA Tesla V100-PCIE-32GB machines are used to run the programs.

**Fig. 2:**
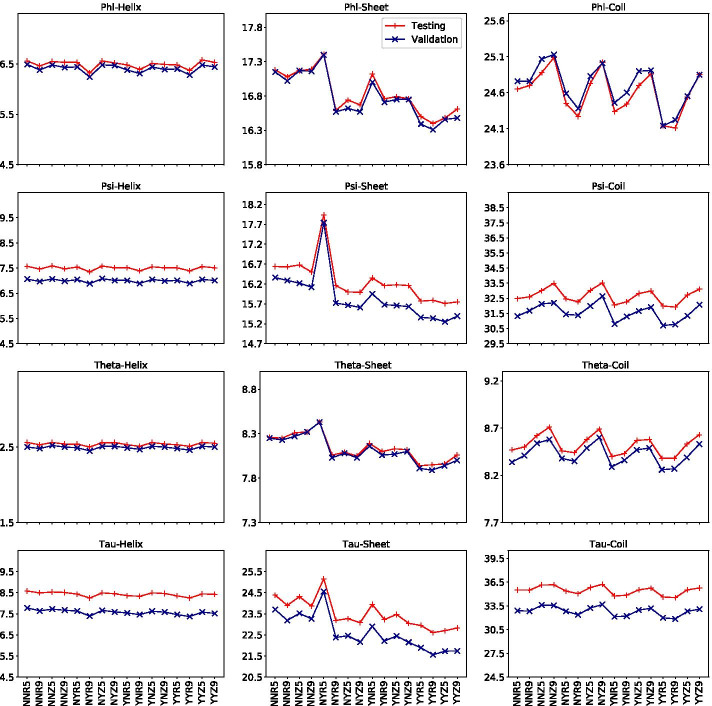
Validation and testing performances in MAE (y-axis) of total 16 SAP4SS settings (x-axis) for each type of backbone angles and for each type of 3-state SS classes

### Benchmark datasets

In this study, we use datasets SPOT-1D [7, 18], PDB150 [19], and CAMEO93 [20]. Our training and validation proteins come from SPOT-1D while testing proteins come from all three datasets. SPOT-1D dataset has proteins that were culled from PISCES [17] on Feb 2017 with the constraints of resolution ($<2.5A^\circ$), R-free $<1$, and a sequence identity cutoff of 25% according to BlastClust [10]. SPOT-1D also has proteins that were released between January 01, 2018 and July 16, 2018 and resolution $< 2.5A^\circ$, R-free $< 0.25$, and 25% sequence identity cutoff w.r.t. those structures released prior to 2018. PDB150 dataset has proteins released between February 1, 2019 and May 15, 2019. For each PDB150 protein, PSI-BLAST [10] was applied against the whole CullPDB [17] dataset with e-value smaller than 0.005. The CAMEO93 dataset contains proteins released between February 2020 and March 2020. We use SSpro8 [28] to generate SS predictions. So we perform 25% sequence similarity checking of training, validation, and testing proteins w.r.t. SSpro8’s training proteins. Moreover, as is done in SAP, we have performed additional filtering to deal with sequence mismatch between data source files and having discontinuity in proteins’ amino acid or secondary structure sequence. Also, we have excluded some proteins that cannot be dealt with by OPUS-TASS and have included large parts of some proteins that have discontinuity. After all these, we have 61 and 55 proteins left in PDB150 and CAMEO93 data sets respectively. Since these are considerably small numbers, we combine these with the testing proteins from SPOT-1D. Table 1 shows the numbers of proteins in each of the training, validation, and testing datasets.

**Table 1:** Numbers of proteins and residues in training, validation, and testing datasets. The testing set comprises 1205, 61, 55 proteins from SPOT-1D, PDB150, CAMEO93 test sets respectively

Datasets	Training	Validation	Testing	Total
Proteins	6721	667	1321	8709
Residues	1,670,605	165,530	306,608	2142,743

### SS specific DNNs

We use 3-state SS predictions made by SSpro8 [28]. These are denoted by *coils*, *helixes*, and *sheets*. We then categorise residues in the training proteins based on the three types. For each of the three types, we then train a separate DNN on the residues that belong to that SS type. For testing, based on which of the three SS types a residue belongs to, we use the particular DNN for the SS type. Table 2 shows the distribution of the residues over the SS types: the top part shows the Dictionary of Protein Secondary Structure (DSSP) based actual classifications and the bottom part shows the predicted classifications.

**Table 2:** Distribution of protein residues over 3-state secondary structure types

SS type	Training		Validation		Testing	
	Residues	Percent	Residue	Percent	Residue	Percent
*Using SS types of residues as in DSSP files*						
Helix	637,203	38.14	61,814	37.34	116,691	38.06
Sheet	383,140	22.93	38,401	23.20	68,975	22.50
Coil	650,262	38.92	65,315	39.46	120,942	39.45
*Using SS types of residues as predicted by SSpro8*						
Helix	637,996	38.19	61,925	37.41	116,568	38.02
Sheet	384,423	23.01	38,723	23.39	69,303	22.60
Coil	648,186	38.80	64,882	39.20	120,737	39.38

## Results

We compare various SAP4SS settings to find the best setting for each of the three SS types and for each of the four angle types. We then compare the performances of the best settings with that of the current state-of-the-art predictors. We also analyse the results in various ways.

### Determining best settings

We consider SAP as our base line setting in which 8-state SS predictions, PSSM, and 7PCP are used as input features and an FCNN with 3 hidden layers as the DNN. For SAP4SS, we consider ASA and HMM as two other types of features. For input encoding, we consider either range based or Z-score based encoding and for window size, we consider either 5 or 9. We, therefore, have 16 SAP4SS settings for each of the three SS types. Each SAP4SS setting is denoted by a name AHIW where A $\in$ {Y, N} denotes whether ASA is used or not, H $\in$ {Y, N} denotes HMM features are used or not, I $\in$ {R, Z} denotes whether range based or Z-score based input encoding is used, and W $\in$ {5, 9} denotes the window size. For example, a setting YNR5 denotes ASA is used, HMM features are not used, range-based input encoding is used, and window size used is 5. Figure 2 shows the validation and testing performances of total 16 SAP4SS settings for each type of backbone angles and for each type of 3-state SS classes. From the charts in the figure, the best settings as shown in Table 3 are selected putting more emphasis on the testing performances and breaking ties with a view to reducing the total number of best settings. Notice that the testing performances in the charts are often worse than the validation performance and for the selected best settings the differences are up to 2.68 for $\tau$ and coils. This is explainable since our testing set is diverse as it include proteins from SPOT-1D, CAMEO93, and PDB150 datasets.

**Table 3:** The best SAP4SS settings for SS types and angle types

SS type	Angle types	Best setting
Helix	$\phi ,\psi ,\theta ,\tau$	YYR9
Sheet	$\phi ,\theta ,\tau$	YYR9
	$\psi$	YYZ9
Coil	$\phi ,\theta$	YYR5
	$\psi ,\tau$	YYR9

For the best settings, we perform 10-fold cross validation to check the robustness of the selected models. The variations in the performances of the 10-fold runs are statistically not significant; so we do not show them.

Hence forth, for further experiments, we use the best settings in Table 3 as the final SAP4SS method and compare it with existing such methods.

### Comparison with existing predictors

We compare SAP4SS’s performance with that of SPOT-1D [7], OPUS-TASS [8], and SAP [30] on our 1321 testing proteins. As described in Section titled Benchmark Datasets, the testing proteins are from SPOT-1D, PDB150, and CAMEO93 datasets.

Table 4 shows the MAE values for various angles as predicted by SAP4SS and existing state-of-the-art methods for residues of various 3-state actual SS types. SAP4SS is better than the existing methods in all cases, except in $\phi$ angles for sheets. To see the relative improvements in MAE values obtained by SAP4SS compared to the existing methods, we compute $\textsf {improvement} = \dfrac{\textsf {2nd Best MAE} - \textsf {SAP4SS MAE}}{\textsf {SAP4SS MAE}}$ (positive value) where SAP4SS obtains the best performance and $\textsf {improvement} = \dfrac{\textsf {Best MAE} - \textsf {SAP4SS MAE}}{\textsf {SAP4SS MAE}}$ (negative value and hence degradation) where SAP4SS is outperformed. From the table, we see the improvements could be more than 6% and in 8 out of 12 cases, more than 1%.

**Table 4:** MAE values for various angles as predicted by various methods for residues of various 3-state actual SS types in our1321 testing proteins

SS-type	Residues	Method	$\phi$	$\psi$	$\theta$	$\tau$
Helix	116,691	SPOT-1D	7.51	11.77	3.36	11.99
		OPUS-TASS	7.10	11.02		
		SAP	6.36	8.14	2.60	9.00
		SAP4SS	**6.31**	**8.08**	**2.53**	**8.73**
		**Improvement**	0.79%	0.74%	2.77%	3.09%
Sheet	68,975	SPOT-1D	16.43	17.85	8.19	23.47
		OPUS-TASS	**15.93**	17.29		
		SAP	17.22	16.68	8.32	24.44
		SAP4SS	16.48	**15.69**	**7.95**	**22.58**
		**Improvement**	− 3.34%	6.31%	3.02%	3.94%
Coil	120,942	SPOT-1D	24.85	37.63	9.28	40.27
		OPUS-TASS	24.33	36.78		
		SAP	24.65	31.99	8.48	35.03
		SAP4SS	**24.18**	**31.30**	**8.37**	**34.09**
		**Improvement**	0.62%	2.20%	1.31%	2.76%

The emboldened values are the best values over the methods compared

Table 5 shows the MAE values for angles as predicted by various methods. SAP4SS performs better than the existing methods in all four types of angles. We compute the relative improvement using the same formula as described before and see that SAP4SS obtains more than 1.5% and less than 4.1% improvement in the MAE values.

**Table 5:** MAE values for angles as predicted by various methods for 306608 residues in 1321 test proteins

SS-type	Residues	Method	$\phi$	$\psi$	$\theta$	$\tau$
All	306,608	SPOT-1D	16.30	23.25	6.76	25.56
Three		OPUS-TASS	15.83	22.49		
Types		SAP	15.96	19.39	6.19	22.60
		SAP4SS	**15.59**	**18.87**	**6.03**	**21.71**
		**Improvement**	1.54%	2.76%	2.65%	4.10%

The emboldened values are the best values over the methods compared. Residues are not categorised using SS types

To check the correlations between the actual angles and the angles predicted by SAP4SS and the existing methods, we compute Spearman rank correlation coefficients and show them in Table 6. As we see, SAP4SS obtains the best positive coefficients among all the methods compared.

**Table 6:** Spearman rank correlation coefficients for the association between actual angles and angles predicted by various methods

Method	$\phi$	$\psi$	$\theta$	$\tau$
SPOT-1D	0.722	0.758	0.779	0.515
OPUS-TASS	0.741	0.767		
SAP	0.731	0.773	0.810	0.518
SAP4SS	**0.745**	**0.780**	** 0.818**	**0.532**

The emboldened values are the best values as the higher the coefficients the better the correlation

**Fig. 3:**
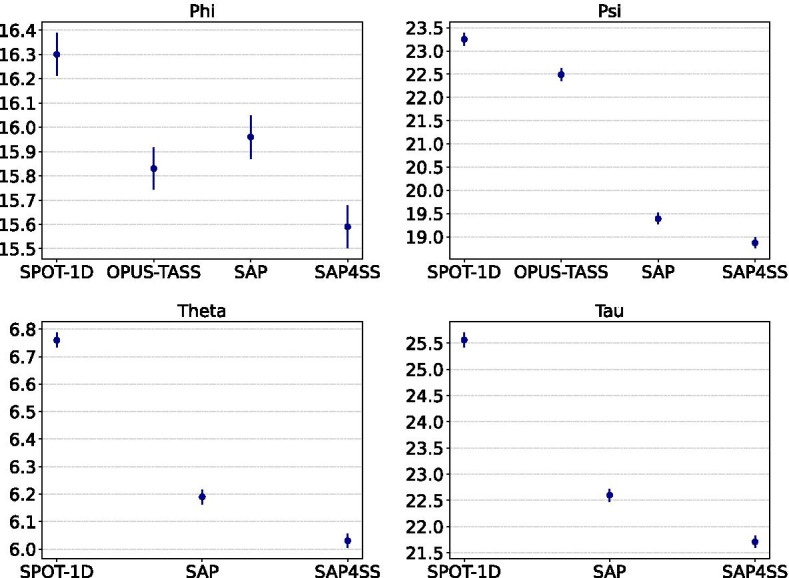
95% confidence intervals of AE values (y-axis) for various methods (x-axis)

To check the statistical significance of the differences in the AE values for various methods, we perform Analysis of Variance (ANOVA) method and for 95% confidence level, we see that at least one method is significantly different from other methods. So as a posthoc analysis technique, we then perform Tukey’s Honest Significant Difference (HSD) to check pairwise difference with 95% confidence level. From the results, we see that the null hypothesis is not rejected only for predictions of $\phi$ values by OPUS-TASS and SAP. For other angles and other pairs of methods, the differences are significant. Figure 3 shows the 95% confidence intervals for the AE values for various methods. Any overlapping e.g. for $\phi$ for OPUS-TASS and SAP denotes the difference is not significant while non-overlapping intervals indicate significant differences.

### Comparison on protein length groups

We group our testing proteins on their numbers of residues and then compare the MAE values of SAP4SS, SAP, OPUS-TASS, and SPOT-1D for each group. For convenience of comparison, in Table 7, MAE values for SAP4SS are shown and for other methods, relative MAEs are computed and shown in $\Delta \%$ columns (formula shown in the caption of the table). As we see, SAP4SS outperforms the other three methods in all cases except one (OPUS-TASS in $\phi$ for length group 301–400).

**Table 7:** Performance of SAP4SS, SAP, OPUS-TASS (in columns OTASS), and SPOT-1D (in columns SPOT) when our testing proteins are grouped based on their lengths

Testing proteins		$\phi$				$\psi$				$\theta$			$\tau$		
		SAP4SS	SAP	OTASS	SPOT	SAP4SS	SAP	OTASS	SPOT	SAP4SS	SAP	SPOT	SAP4SS	SAP	SPOT
Length	Count	MAE	$\Delta$%	$\Delta$%	$\Delta$%	MAE	$\Delta$%	$\Delta$%	$\Delta$%	MAE	$\Delta$%	$\Delta$%	MAE	$\Delta$%	$\Delta$%
001–100	232	14.60	+ 2.05	+ 1.03	+ 3.77	18.47	+ 1.08	+ 15.54	+ 17.16	5.63	+ 1.95	+ 9.59	20.39	+ 3.68	+ 13.68
101–200	424	15.32	+ 2.48	+ 1.17	+ 3.98	18.78	+ 2.50	+ 15.65	+ 19.33	6.05	+ 2.64	+ 9.75	22.01	+ 4.27	+ 14.08
201–300	294	15.24	+ 2.49	+ 2.23	+ 4.66	18.41	+ 2.66	+ 19.77	+ 23.19	5.95	+ 2.35	+ 11.93	22.00	+ 3.27	+ 16.55
301–400	190	15.60	+ 2.88	− 0.19	+ 3.53	18.58	+ 3.39	+ 16.85	+ 22.50	6.04	+ 2.98	+ 11.42	21.23	+ 5.37	+ 17.57
401–500	103	15.80	+ 2.09	+ 3.92	+ 7.28	18.57	+ 2.85	+ 27.95	+ 31.07	5.96	+ 2.52	+ 16.61	20.75	+ 4.14	+ 24.24
501–800	78	16.64	+ 1.98	+ 1.08	+ 4.21	20.59	+ 2.77	+ 19.14	+ 23.75	6.34	+ 2.68	+ 12.93	22.96	+ 3.48	+ 19.99
Overall	1321	15.59	+ 2.37	+ 1.54	+ 4.55	18.87	+ 2.76	+ 19.18	+ 23.21	6.03	+ 2.65	+ 12.11	21.71	+ 4.10	+ 17.73

In the table, $\Delta$% of a method (e.g. SAP, OPUS-TASS, or SPOT-1D) is computed as $\dfrac{\textsf {its MAE} - \textsf {SAP4SS MAE}}{\textsf {SAP4SS MAE}}*100\%$. The greater the value of $\Delta$MAE, the worse the performance of the method w.r.t. SAP4SS

### Correct prediction per protein

**Fig. 4:**
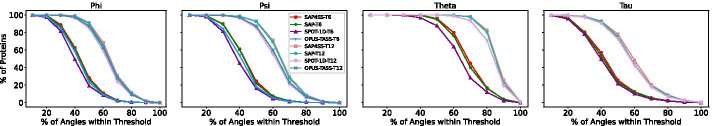
Percentages of proteins (y-axis) having certain percentages of angles (x-axis) with AE within threshold 6 (T6) and 12 (T12) degrees. The lower the threshold, the better the results

We compare SAP4SS with SAP, OPUS-TASS, and SPOT-1D on the percentages of proteins having certain percentages of angles correctly predicted within given threshold MAE. The threshold MAE values are 6$^{\circ }$ and 12$^{\circ }$; in SAP and SPIDER, multiples of 6$^{\circ }$ have been used as thresholds and the lowest MAE is about 6$^{\circ }$ for one angle e.g. $\theta$. The percentages of angles are varied from 0 to 100% with step 10%. Figure 4 shows these results. We see that with higher percentages of proteins having the same percentages of angles within thresholds, SAP4SS outperforms in all angles in both threshold levels.

### Protein structure generation

**Fig. 5:**
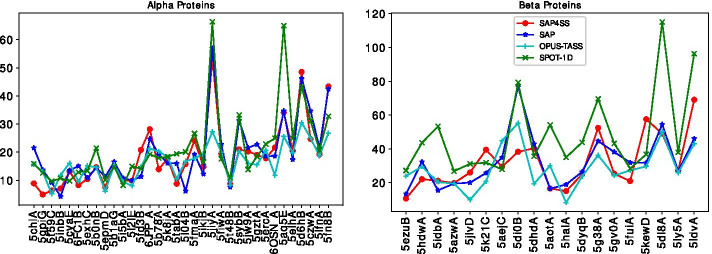
RMSD values (y-axis) of protein structures (x-axis) generated using $\phi$ and $\psi$ angles predicted by various methods. In the charts proteins are sorted in the x-axis based on their lengths

With the $\phi$ and $\psi$ angles predicted by various methods compared, we generate the protein structures for a number of proteins. From our 1321 proteins, we take only the whole proteins but not the domains. Then, we create a subset named *alpha proteins* taking 34 proteins that have at least $60\%$ helix residues. Similarly, we create a subset named *beta proteins* taking 19 proteins that have at least $60\%$ sheet residues. While generating the protein structures $\omega$ angles are assumed to be $180^\circ$ and *C*-*N* peptide bonds are assumed to have length 1.33Å. The bond angles and bond lengths are usually standard within different residues of the same amino acids over native structures of various proteins. Using the standard bond angles and bond lengths, we could build each amino acid residue from scratch. However, we take an alternative way, in which we take one residue for each amino acid from native structures of some other known proteins and build a residue library. Each of the 20 residues in the library essentially has coordinates for up to $C^\beta$ atoms; note necessary bodily translation and rotation operations are performed to establish local coorindate systems. Concatenating these residues, using each residue as many times as we need for the given protein, we first create a linear chain for a protein and then apply the predicted $\phi$ and $\psi$ angles on each residue to get the generated conformation. Figure 5 shows the RMSD values for various methods for the alpha and beta proteins. The performances are clearly far from a reasonable target of 6Å and any comparison with large RMSD values is merely indicative. Nevertheless, we see that SAP4SS has shown comparable performance with respect to other methods in both alpha and beta proteins. In this context, note that SAP4SS and SAP use sliding windows to capture local interactions while OPUS-TASS and SPOT-1D use entire proteins to capture global interactions.

## Conclusion

In this paper, we improve accuracy of backbone angle prediction for protein structures. Machine learning methods loose accuracy in the process of achieving generality over the training proteins. We aim to supplement our deep learning method with specific knowledge about secondary structure types. Using predicted secondary structure types, we categorise residues and then train separate deep learning models for each category. This essentially restricts the generalisation process within the specific category. Our method named SAP4SS obtains mean absolute error values of 15.59, 18.87, 6.03, and 21.71 respectively for four types of backbone angles $\phi$, $\psi$, $\theta$, and $\tau$. These are 1.5–4.1% better than predictions made by the current state-of-the-art prediction methods. SAP4SS along with its data is available from https://gitlab.com/mahnewton/sap4ss.

## Data Availability

SAP4SS along with its data is available from https://gitlab.com/mahnewton/sap4ss.
